# The long non-coding RNA BBOX1 antisense RNA 1 is upregulated in polycystic ovary syndrome (PCOS) and suppresses the role of microRNA-19b in the proliferation of ovarian granulose cells: Short title: BBOX1 antisense RNA 1 in cell proliferation

**DOI:** 10.1186/s12905-023-02632-5

**Published:** 2023-09-21

**Authors:** Zhi Zhou, Yong Zhang, Can Tan, Juan Zhang, Guohui Yi, Bangbei Wang, Yejuan Li, Hui Lu, Weiying Lu, Xiaopo Zhang

**Affiliations:** 1 Reproductive Medical Center, Hainan Women and Children’s Medical Center, No.75 South Longkun Road, 570206 Haikou City, Hainan Province P.R. China; 2 Department of Pharmacology, School of Basic Medicine and Life Science, Hainan Medical University, 571199 Haikou City, Hainan Province P.R. China; 3 Feinberg Cardiovascular and Renal Research Institute, Feinberg School of Medicine, Northwestern University, 60611 Chicago, IL USA; 4 Reproductive Medical Center, Zhuzhou Central Hospital, 412007 Zhuzhou City, Hunan Province P.R. China; 5 Public Research Laboratory, Hainan Medical University, 571199 Haikou City, Hainan Province P.R. China; 6 Key Laboratory of Tropical Translational Medicine of the Ministry of Education, Hainan Key Laboratory for Research and Development of Tropical Herbs, School of Pharmacy, Hainan Medical University, 571199 Haikou City, Hainan Province P.R. China

**Keywords:** Polycystic ovary syndrome, miR-19b, BBOX1-AS1, Granulosa cell proliferation

## Abstract

**Background:**

MicroRNA-19b (miR-19b) has been reported to be downregulated in polycystic ovary syndrome (PCOS), while its upstream regulators are unclear. We speculated that miR-19b could potentially form a binding relationship with BBOX1 antisense RNA 1 (BBOX1-AS1), a long non-coding RNA recognized for its critical role in ovarian cancer. Subsequently, we investigated into their interaction in PCOS.

**Methods:**

The expression of miR-19b and BBOX1-AS1 in follicular fluid from both control women (n = 80) and women with PCOS (n = 80) was detected by RT-qPCR. Correlations were analyzed with Pearson’ correlation coefficient. The binding of miR-19b to the wild-type (-wt) ad mutant (-mut) BBOX1-AS1 was determined by RNA-RNA pulldown assay. Their interactions were detected by overexpression assay. Bromodeoxyuridine (BrdU) assay was applied for proliferation analysis.

**Results:**

BBOX1-AS1 was highly upregulated, while miR-19b was downregulated in PCOS. There was no close correlation across PCOS and the control samples. Consistently, they did not regulate the expression of each other in granulosa cells. However, BBOX1-AS1-wt, but not BBOX1-AS1-mut, could directly interact with miR-19b. BBOX1-AS1 suppressed the role of miR-19b in inhibiting granulosa cell proliferation.

**Conclusion:**

BBOX1-AS1 is highly upregulated in PCOS, and it may serve as an endogenous competing RNA for miR-19b to suppress its role in inhibiting granulosa cell proliferation. Our study suggested the role of BBOX1-AS1 as a potential target to treat PCOS.

**Supplementary Information:**

The online version contains supplementary material available at 10.1186/s12905-023-02632-5.

## Background

Polycystic ovary syndrome (PCOS) is a common clinical disorder in the reproductive organ ovary that affects about 4–12% of women at their reproductive age [1]. In China, the estimated overall incidence of PCOS stands at approximately 7.8% [2]. Hormone disorders are common in PCOS. In effect, PCOS is characterized by the increased gonadotropin-releasing hormone (GnRH), which can cause increased secretion of luteinizing hormone (LH) from the pituitary gland. High levels of LH results in high levels of androgens, leading to decreased levels of follicle-stimulating hormone (FSH). Consequently, poor egg development and an inability to ovulate will happen [1, 2]. Moreover, Ovarian steroidogenesis involved in PCOS affects the production of estrogen and androgen, or even progesterone. PCOS is also characterized by the formation of numerous small fluid-filled sacs or cysts in the ovary [3]. Without proper treatment, PCOS may cause reduced egg quality and prevent ovulation, leading to infertility [4]. To date, the cause of PCOS is unknown. However, increased levels of male hormones may contribute to the initiation and progression of PCOS by preventing the production of hormones in the ovary and the formation of eggs [5]. Although PCOS is treatable and pregnancy can usually be achieved in most cases, there is no cure for PCOS and lifelong treatment is required [6, 7]. In the United States, the treatment and diagnosis of PCOS incur costs exceeding 8 million dollars [8].

Extensive efforts have been made to treat PCOS, such as the use of Galega officinalis and the extracts of Apium graveolens and Cinnamon zeylanicum to treat animal models of PCOS [9, 10]. However, these novel approaches are still under research. Despite the unknown causes of PCOS, a considerable number of molecular players have been characterized in this clinical disorder [11]. Certain molecular factors with a pivotal role in PCOS have been demonstrated to be potential targets to treat PCOS [12, 13]. Non-coding RNAs (ncRNAs), such as long ncRNAs (lncRNAs) and microRNAs (miRNAs), do not contain coding information of proteins but interact with proteins, DNAs and other RNAs to participate in the regulating of protein accumulation, gene expression, and chromatin stability, thereby are critical regulators of diseases [14, 15]. Therefore, lncRNAs may serve as the next-generation targets of PCOS. For instance, the progression of PCOS can be controlled through the regulation of lncRNAs placenta-specific protein and HLA-F antisense RNA 1 [15, 16]. MiR-19b has been reported to be downregulated in PCOS [17], suggesting its potential involvement in this disease. We predicted that miR-19b could bind to BBOX1 antisense RNA 1 (BBOX1-AS1), a lncRNA with a critical role in ovarian cancer [18]. Therefore, BBOX1-AS1 may interact with miR-19b to participate in PCOS. This study was then carried out to investigate their interaction in PCOS.

## Materials and methods

### Patients and samples

Follicular fluid samples were donated by 80 patients with PCOS (29.6 +/- 4.1 years old) and 80 controls (29.3 +/- 4.1 years old) at Hainan Women and Children’s Medical Center after the Ethics Committee of this hospital approved this study. All participants signed the informed consent, who underwent their first round of in-vitro fertilization treatment. Sample collection was performed during in vitro fertilization (IVF) through intracytoplasmic injection. Controls in the present study received IVF treatment due to male factors. Rotterdam revised criteria were followed to diagnose PCOS [19]. Participants older than 40 years old and patients with a body mass index higher than 35 were excluded.

### Cells and cell culture method

Gene interactions and the functional roles of genes were assessed through in vitro cell assays employing human ovarian granulosa cell-like KGN cells (ATCC). Subculture was carried out in a 10:1 volume ratio when cell confluent reached approximately 70 and 80%. Cells were cultivated in 6 cm plates using DMEM supplemented with 10% FBS. In instances of cell contamination, the affected cell culture plates were discarded, and the cell culture process was repeated.

### Cell transfection

By means of transfection with BBOX1-AS1 vector, BBOX1-AS1 siRNA, and miR-19b mimic, we investigated their interactions and roles in modulating cellular behaviors. To outline the process, cells were harvested, rinsed with ice-cold PBS, and then quantified. Then cells were transferred to a tube containing transfection mixture (Lipofectamine™ 3000 and vector, siRNA or miRNA). After incubation for 6 h, cells were washed for three times with PBS and transferred to 6 cm cell culture plates to reduce cytotoxicity.

### RNA isolation

For RNA isolation, Trizol (Invitrogen) was combined with samples in a 10:1 volume ratio. After incubating at room temperature for 30 min, centrifugation (12,000 g) was carried out for 12 min to eliminate cellular debris. Subsequently, the supernatant was collected, and chloroform was mixed in a 1:4 volume ratio to eliminate protein contamination. After mixing, another centrifugation step (12,000 g) was conducted for 12 min. The supernatant was then collected and combined with methanol at a 1:1 volume ratio, followed by centrifugation (12,000 g) to precipitate the RNA samples.

### RT-qPCR

RNase-free water was used to dissolve RNA samples. RNA concentration, purity, and integrity were determined using a 2100 bioanalyzer. After cDNA preparation, PCR amplification of GAPDH was performed on each cDNA sample to determine cDNA quality. Samples with satisfactory quality were used to perform qPCRs with 18S rRNA as the internal control to determine the expression levels of BBOX1-AS1 and miR-19b. Poly (A) addition was performed before the detection of miR-19 expression. In cases of the failure of GAPDH amplification, cDNA preparation and/or RNA preparation were repeated. PCR reactions were carried out on a Bio-Rad CFX96 qPCR Real-Time PCR Module (Bio-Rad). The 2^−ΔΔCT^ method was used for normalization of Ct values. Primer sequences were: 5’-TGTGTGTTTCCTGAGGCCTC-3’ (forward) and 5’-CGCCTCTCTTGGAACACCTT-3’ (reverse) for BBOX1-AS1; 5’-GTAACCCGTTGAACCCCATT-3’ (forward) and 5’-CCATCCAATCGGTAGTAGCG-3’ (reverse) for 18S rRNA; 5’-TGTGCAAATCCATGCAAAAC-3’ (forward) and Oligo d (T) for miR-19b.

### RNA-RNA pull-down

RNA-RNA pull-down was performed as previously described [20]. Briefly, Biotin-labeled RNAs, including NC (Bio-NC), BBOX1-AS1 wild type (Bio-BBOX1-AS1-wt), and BBOX1-AS1 mutant (Bio-BBOX1-AS1-mut) were prepared by performing in vitro transcriptions with T7 in vitro transcriptase, followed by biotin labeling. After transfecting the cells with labeled RNAs, cell culture was carried out for 48 h, then cells were collected, and cell lysis was performed for 30 min on ice. RNA pulldown was performed by incubating cell lysates with streptavidin agarose resin beads. After the collection of beads, RNA complexes were purified and miR-19b accumulation was determined by performing RT-PCR.

### Subcellular fractionation assay

Nuclear and cytoplasmic fractions were prepared from KGN cells using the NE-PER Nucleus and Cytoplasmic Extraction Reagents (Thermo Fisher Scientific). All operations were completed following the manufacturers’ instructions. Briefly, cells were used to prepare cell lysate, and cytoplasmic fraction was isolated through centrifugation. Nuclear fraction, which was the pellet, was subjected to nucleus lysis. Two fractions were then used to isolate RNA, which was subjected to RT-PCR to determine the expression of BBOX1-AS1.

### BrdU assay

BrdU assay was performed as previously described [21]. Briefly, cells were collected, washed with PBS, and counted. Subsequently, cell culture was carried out in a 24-well plate, and BrdU (10 µg/mL) was introduced 46 h after initiating the cell culture. Following a 2-h incubation period, a peroxidase-coupled BrdU antibody was added and incubated for an additional 2 h. Then, tetramethylbenzidine, the substrate of the antibody was added, followed by incubation for 30 min. Finally, OD values at 450 nm were measured to determine cell proliferation.

### Statistical analyses

Statistical power was calculated using GraphPad Prism 6 software and a statistical power > 0.85 was achieved in all cases. Student’s t-test was used to explore the differences between the 2 groups. Multiple groups were compared by ANOVA Tukey’s test. Data comparisons and image preparations were performed with SPSS software 21 (SPSS Inc). A *p*-value smaller than 0.05 indicated a difference with statistical significance.

## Results

### The expression of miR-19b and BBOX1-AS1 in PCOS

**Fig. 1:**
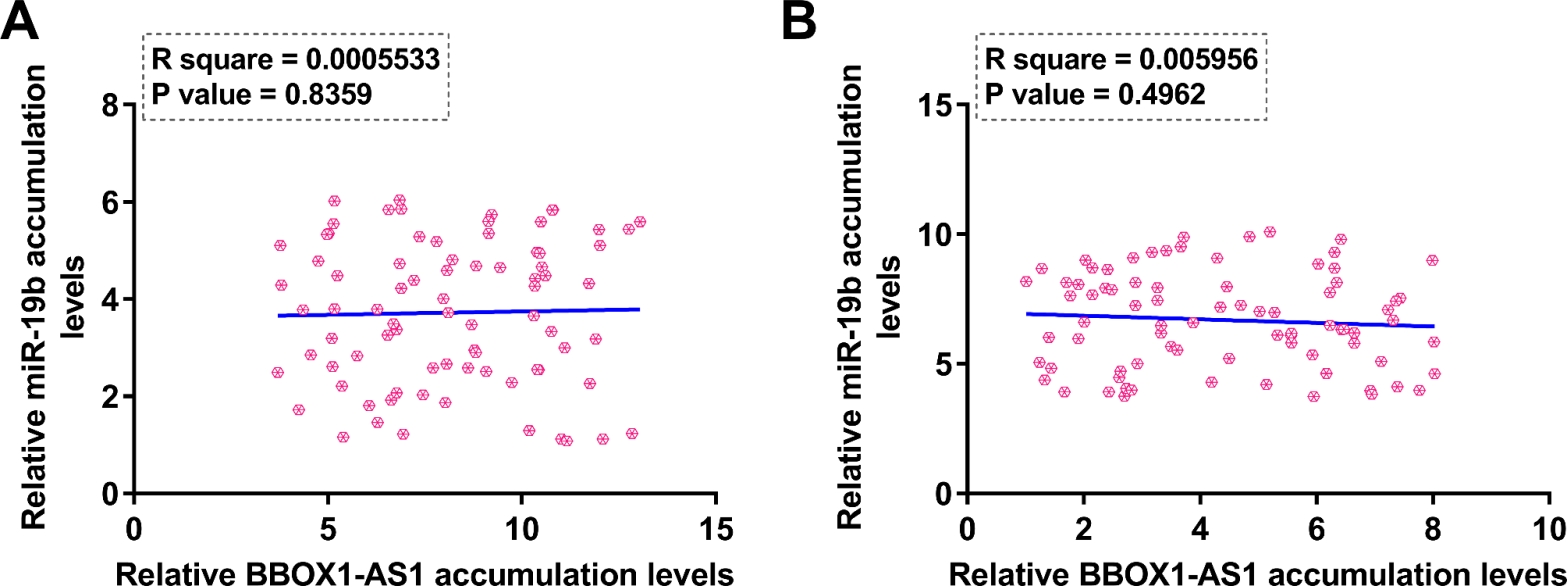
Analysis of miR-19b and BBOX1-AS1 expression in PCOS RNA accumulation determines its function. To this end, accumulation of BBOX1-AS1 (**A**) and miR-19b (**B**) in follicular fluid from both control women (n = 80) and women with PCOS (n = 80) was analyzed with RT-qPCR. **, *p* < 0.01

The function of RNA hinges on its accumulation level. To this end, the levels of BBOX1-AS1 and miR-19b accumulation in follicular fluid were assessed using RT-qPCR from both control women (n = 80) and women with PCOS (n = 80) to speculate the function of BBOX1-AS1 and miR-19b in PCOS. BBOX1-AS1 was highly upregulated in PCOS (Fig. 1A, p < 0.01), while miR-19b was significantly downregulated (Fig. 1B, p < 0.01). Therefore, increased expression levels of BBOX1-AS1 and decreased expression levels of miR-19b may contribute to PCOS, or they are the consequences of PCOS.

### Correlation analysis between miR-19b to BBOX1-AS1

**Fig. 2:**
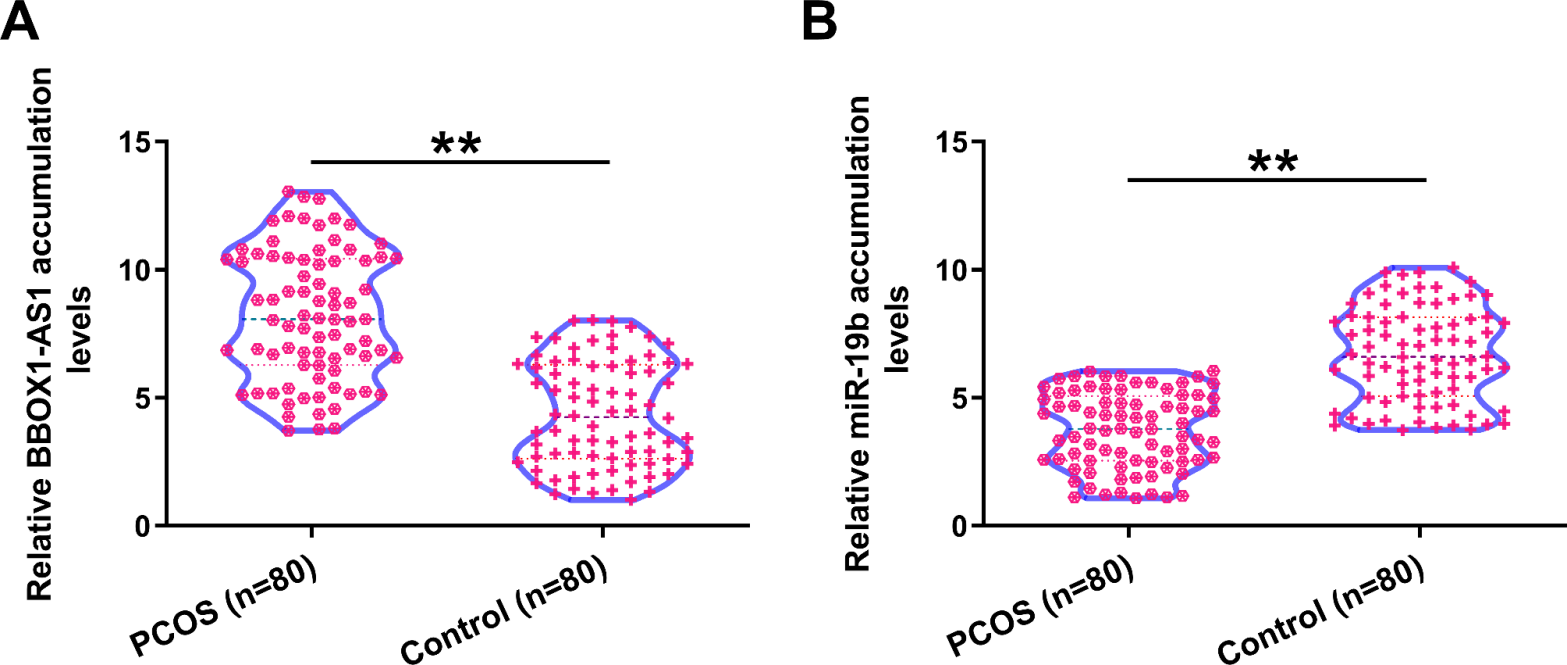
Correlation analysis between miR-19b to BBOX1-AS1 Correlations between miR-19b to BBOX1-AS1 across PCOS (**A**) and control (**B**) samples were studied with Pearson’ correlation coefficient

Correlation analysis could indicate interactions between genes. Therefore, correlations between miR-19b and BBOX1-AS1 across PCOS and control samples were studied with Pearson’s correlation coefficient. However, no close correlation was detected across PCOS (Fig. 2A) and control (Fig. 2B) samples. Therefore, miR-19b and BBOX1-AS1 may not regulate the expression of each other.

### The binding of miR-19b to BBOX1-AS1

**Fig. 3:**
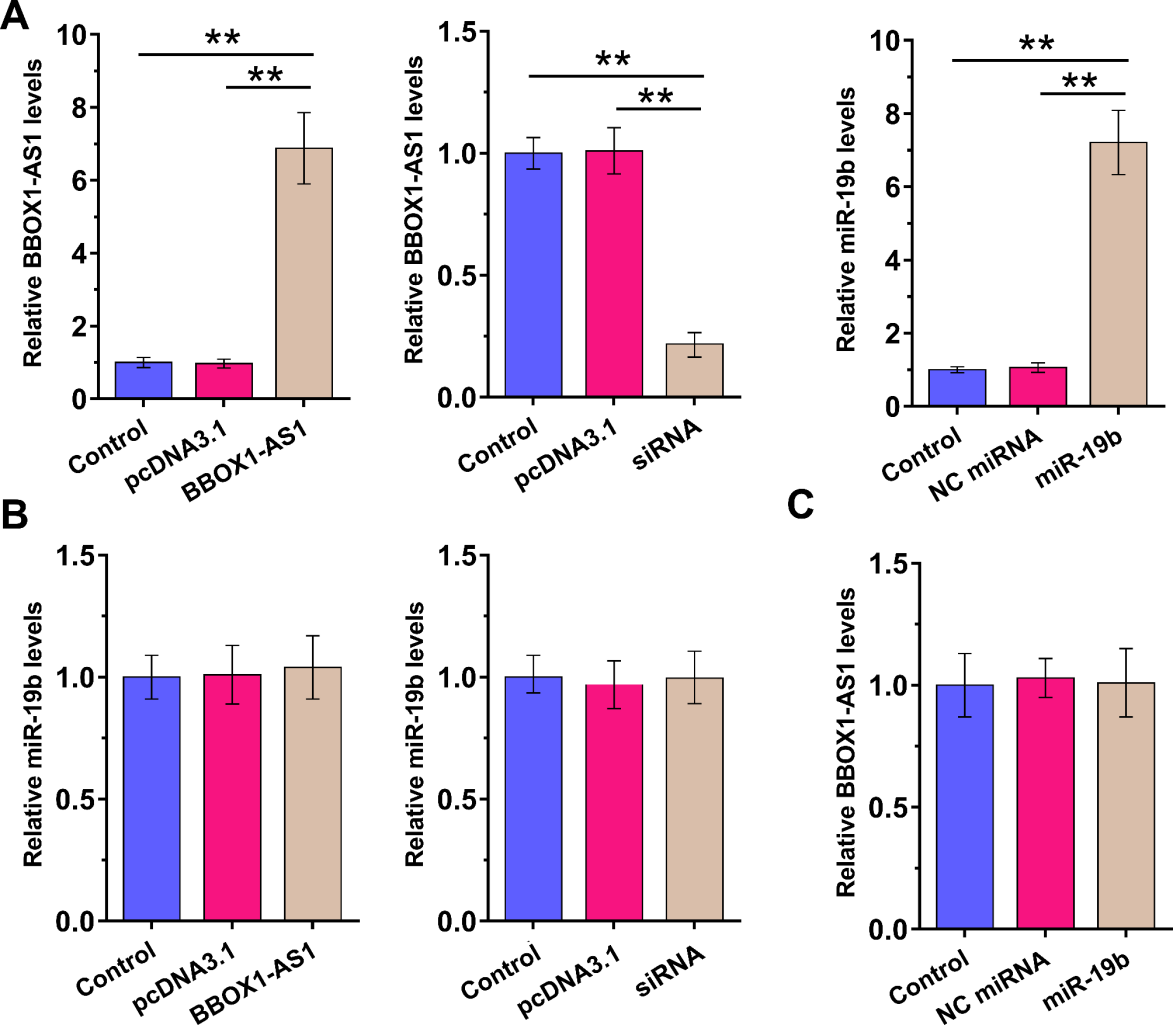
The binding of miR-19b to BBOX1-AS1 The binding of miR-19b to BBOX1-AS1 was predicted by IntaRNA 2.0 (**A**). To confirm this prediction, the binding of miR-19b to BBOX1-AS1 wild type (-wt, **B**) ad mutant (-mut, **C**) was determined by RNA-RNA pulldown assay. Subcellular fractionation assay was conducted to determine the subcellular location of BBOX1-AS1 (**D**). **, *p* < 0.01

The binding of miR-19b to BBOX1-AS1 was predicted by IntaRNA 2.0, which showed that miR-19b and BBOX1-AS1 may form a binding relationship with each other (Fig. 3A). To confirm this, the binding of miR-19b to the wild type of BBOX1-AS1 (-wt) and mutant (-mut) in KGN cells was determined by RNA-RNA pulldown assay. It showed that BBOX1-AS1-wt (Fig. 3B, p < 0.01), but not BBOX1-AS1-mut (Fig. 3C), directly interacted with miR-19b. Therefore, BBOX1-AS1 may only serve as an endogenous competing RNA for miR-19b but did not regulating its expression. Subcellular fractionation assay was conducted to determine the subcellular location of BBOX1-AS1. It was observed that, different from GAPDH, which is a cytoplasmic marker, BBOX1-AS1 can be detected in both cytoplasm and nucleus of KGN cells (Fig. 3D).

### Regulatory role of BBOX1-AS1 and miR-19b in regulating the expression of each other

**Fig. 4:**
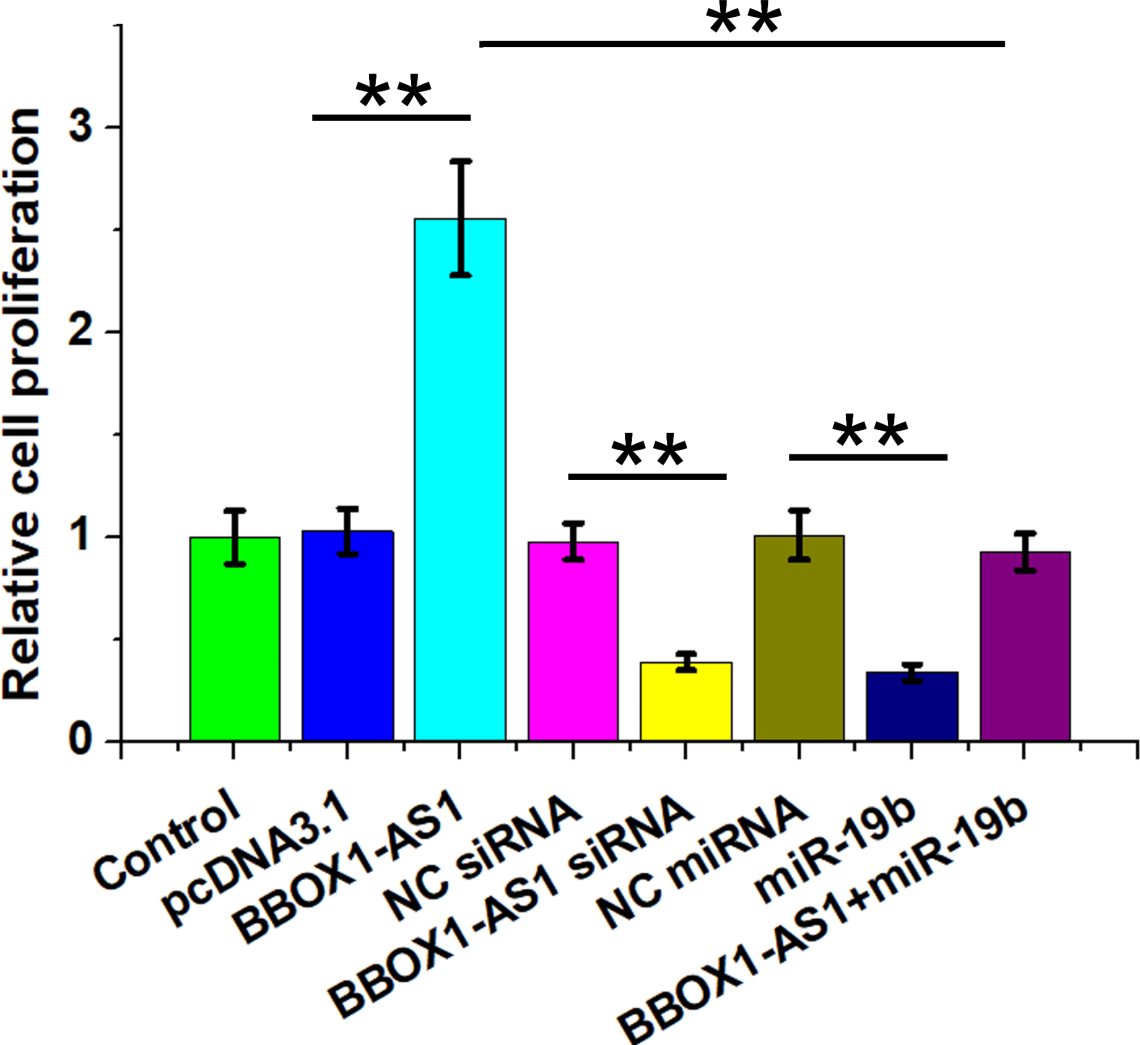
Regulatory role of BBOX1-AS1 and miR-19b in each other’s accumulation KGN cells were overexpressed with BBOX1-AS1 or miR-19b to study their regulatory roles in in each other’s accumulation (**A**). The role of BBOX1-AS1 in miR-19b accumulation (**B**) and the role of miR-19b in BBOX1-AS1 accumulation (**C**) were analyzed by performing RT-qPCR. **, *p* < 0.01

To investigate their regulatory roles in regulating the expression of each other, BBOX1-AS1 or miR-19b was overexpressed in KGN cells (Fig. 4A, p < 0.01). Moreover, silencing of BBOX1-AS1 was also achieved (Fig. 4A, p < 0.01). Consistent with the correlation analysis, overexpression or silencing of BBOX1-AS1 had no effect in regulating the expression of miR-19b (Fig. 4B), and miR-19b did not regulate the expression of BBOX1-AS1 (Fig. 4C). These results further supported the role of BBOX1-AS1 as an endogenous competing RNA for miR-19b.

### Regulatory role of BBOX1-AS1 and miR-19b in regulating KGN cell proliferation

**Fig. 5:**
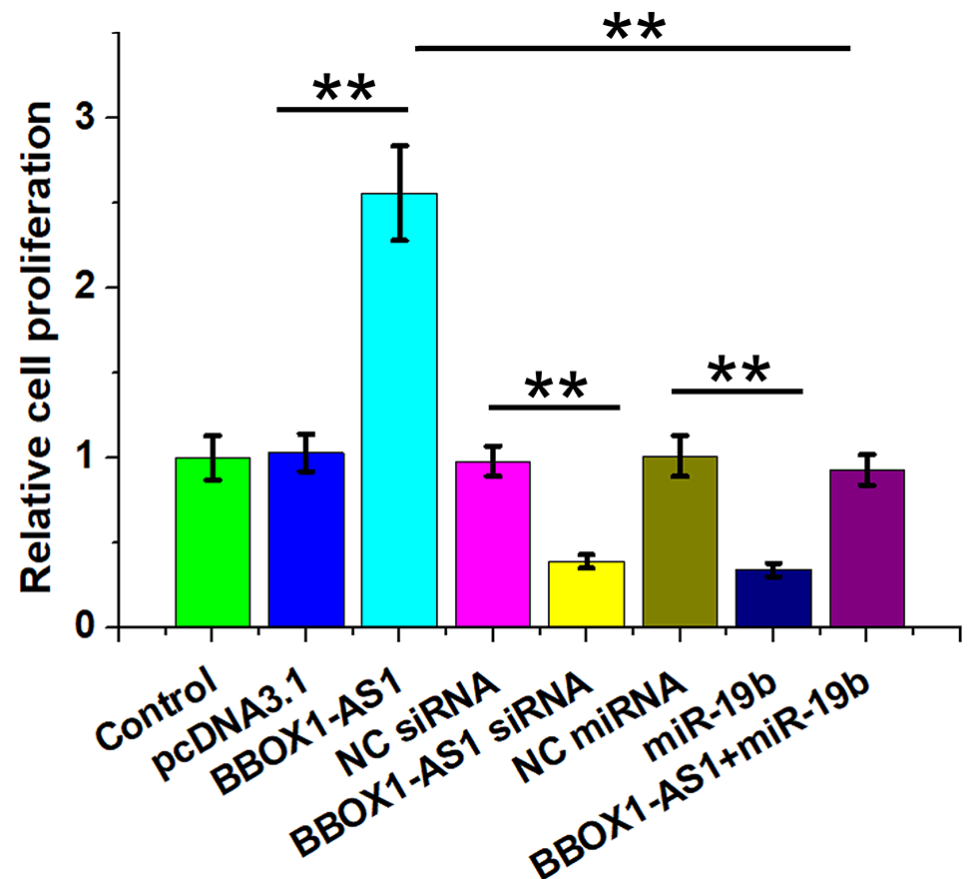
Regulatory role of BBOX1-AS1 and miR-19b in KGN cell proliferation Regulatory role of overexpression and silencing of BBOX1-AS1 as well as overexpression of miR-19b in KGN cell proliferation was studied with BrdU assay. **, *p* < 0.01

Altered cell proliferation contributes to PCOS. To this end, the regulatory role of BBOX1-AS1 and miR-19b in regulating KGN cell proliferation was investigated by conducting BrdU assay. BBOX1-AS1 increased cell proliferation, while overexpression of miR-19b and silencing of BBOX1-AS1 decreased cell proliferation. BBOX1-AS1 suppressed the role of miR-19b in inhibiting KGN cell proliferation (Fig. 5, p < 0.01). Therefore, BBOX1-AS1 may affect cell proliferation in PCOS through regulating miR-19b to participate in this disease.

## Discussion

Evidence has consistently shown that non-coding RNAs (ncRNAs) play pivotal roles in various human diseases. A deeper understanding of the functions of ncRNAs in these conditions holds the potential to offer fresh perspectives for treatment design. The present study explored the involvement of BBOX1-AS1 and miR-19b in PCOS, a common reproductive problem in adult women. We observed the altered expression of BBOX1-AS1 and miR-19b in PCOS. Our findings also demonstrated that BBOX1-AS1 may serve as an endogenous competing RNA for miR-19b to suppress its function in cell proliferation.

A recent study reported the decreased expression levels of miR-19b in patients with PCOS, where miR-19b targets insulin-like growth factor 1 (IGF-1) to promote the proliferation of granulosa cells [17]. PCOS is characterized by increased GnRH pulsatility, which stimulates the proliferation of granulosa cells [1, 2]. The main role of granulosa cells is to produce LH receptors and steroids [22]. In effect, reduced apoptotic rate and increased proliferation rate of granulosa cells were frequently observed in patients with PCOS and may contribute to the progression of this disease [22]. Theca cells autonomously synthesize androgen and progesterone. Granulosa cells convert theca cell-produced androgens into estrogens [1, 2]. In other words, granulosa cells and surrounding theca cells determine ovarian steroidogenesis. Therefore, miR-19b may be targeted to treat PCOS. This study confirmed the decreased accumulation of miR-19b in PCOS and its enhancing effects on the proliferation of granulosa cells. However, the upstream regulator or miR-19b is unclear.

We predicted that miR-19b could bind to BBOX1-AS1, and this prediction was verified by RNA-RNA pull-down assay. Interestingly, these two non-coding RNAs are not correlated, and they do not regulate the expression of each other. BBOX1-AS1 is a critical player in ovarian cancer, where it upregulates PODXL by sponging miR-361-3p, thereby promoting cancer progression [18]. The present study showed the involvement of BBOX1-AS1 in PCOS, which is also a clinical disorder in ovaries. Therefore, BBOX1-AS1 is likely involved in multiple types of ovarian disorders. Interestingly, BBOX1-AS1 suppressed the role of miR-19b in promoting the proliferation of granulosa cells. Therefore, we concluded that BBOX1-AS1 may serve as an endogenous competing RNA for miR-19b to suppress its role. However, this speculation should be further verified.

Our study characterized a novel BBOX1-AS1/miR-19b axis in PCOS. It is known that oxidative stress-induced ovarian tissue damage contributes to the progression of PCOS [23–25]. Therefore, the involvement of BBOX1-AS1/miR-19b in oxidative stress in ovary should be studied in future. Studies may also focus on the clinical application of this novel pathway in the treatment of PCOS.

## Conclusion

BBOX1-AS1 is highly expressed in PCOS, while miR-19b is downregulated in PCOS. Moreover, BBOX1-AS1 may serve as an endogenous competing RNA for miR-19b to suppress its role in inhibiting granulosa cell proliferation, thereby contributing to the progression of PCOS. Our study characterized a novel BBOX1-AS1/miR-19b axis in PCOS, which may serve as a potential target to treat PCOS.

### Electronic supplementary material

Below is the link to the electronic supplementary material.

Supplementary Material 1

Supplementary Material 2

## Data Availability

The datasets used and/or analyzed during the current study are available from the corresponding author on reasonable request.
