# Supplementary material for: The long non-coding RNA BBOX1 antisense RNA 1 is upregulated in polycystic ovary syndrome (PCOS) and suppresses the role of microRNA-19b in the proliferation of ovarian granulose cells: Short title: BBOX1 antisense RNA 1 in cell proliferation
Source: BMC Womens Health. 2023 Sep 21;23:508. doi: 10.1186/s12905-023-02632-5 (PMC10512487; doi:10.1186/s12905-023-02632-5)

“The original figure presented in old Fig.3 has been cropped and saved as the original name. So the original image was replaced by the cropped one. We have another three replicates. Fig.3 has been modified to present the result of one of the three replicates. The orginal image has also been provided”

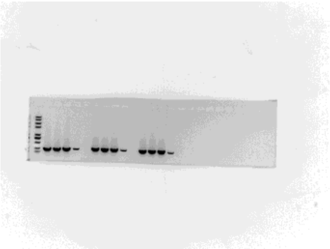

Supplement: Supplementary file 1 — Supplementary Material 1 [file 12905_2023_2632_MOESM1_ESM.pdf]
