# Supplementary figures and images for: The long non-coding RNA BBOX1 antisense RNA 1 is upregulated in polycystic ovary syndrome (PCOS) and suppresses the role of microRNA-19b in the proliferation of ovarian granulose cells: Short title: BBOX1 antisense RNA 1 in cell proliferation
Source: BMC Womens Health. 2023 Sep 21;23:508. doi: 10.1186/s12905-023-02632-5 (PMC10512487; doi:10.1186/s12905-023-02632-5)

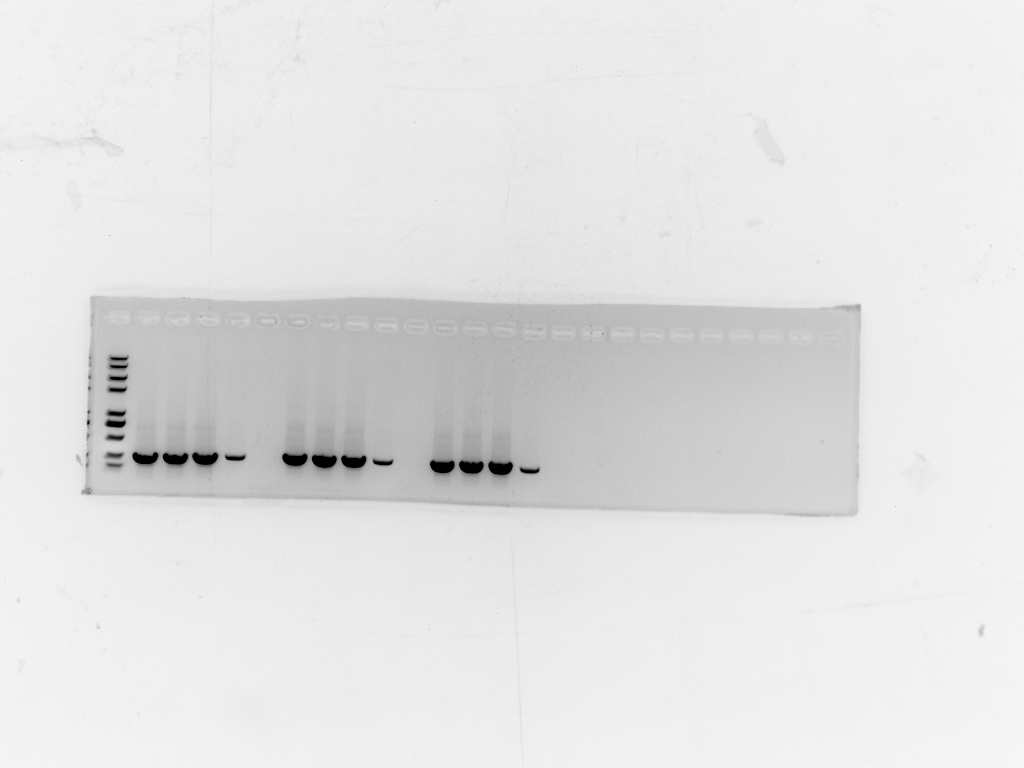

Supplement: Supplementary file 2 — Supplementary Material 2 [file 12905_2023_2632_MOESM2_ESM.png]
